# A qualitative exploration of the barriers and facilitators affecting ethnic minority patient groups when accessing medicine review services: Perspectives of healthcare professionals

**DOI:** 10.1111/hex.13410

**Published:** 2021-12-23

**Authors:** Anna Robinson, Muna Elarbi, Adam Todd, Andy Husband

**Affiliations:** ^1^ School of Pharmacy, Newcastle University Newcastle Upon Tyne UK; ^2^ Institute of Population Health Sciences, Newcastle University Newcastle Upon Tyne UK

**Keywords:** BAME, ethnic minority, ethnicity, medicine review, medicine services, prescribing safety

## Abstract

**Introduction:**

Healthcare inequalities and ethnicity are closely related. Evidence has demonstrated that patients from ethnic minority groups are more likely to report a long‐term illness than their white counterparts; yet, in some cases, minority groups have reported poorer adherence to prescribed medicines and may be less likely to access medicine services. Knowledge of the barriers and facilitators that impact ethnic minority access to medicine services is required to ensure that services are fit for purpose to meet and support the needs of all.

**Methods:**

Semistructured interviews with healthcare professionals were conducted between October and December 2020, using telephone and video call‐based software. Perspectives on barriers and facilitators were discussed. Interviews were audio‐recorded and transcribed verbatim. Reflexive thematic analysis enabled the development of themes. QSR NVivo (Version 12) facilitated data management. Ethical approval was obtained from the Newcastle University Faculty of Medical Sciences Ethics Committee.

**Results:**

Eighteen healthcare professionals were interviewed across primary, secondary and tertiary care settings; their roles spanned medicine, pharmacy and dentistry. Three themes were developed from the data regarding the perceived barriers and facilitators affecting access to medicine services for ethnic minority patients. These centred around patient expectations of health services; appreciating cultural stigma and acceptance of certain health conditions; and individually addressing communication and language needs.

**Conclusion:**

This study provides much‐needed evidence relating to the barriers and facilitators impacting minority ethnic communities when seeking medicine support. The results of this study have important implications for the delivery of person‐centred care. Involving patients and practitioners in coproduction approaches could enable the design and delivery of culturally sensitive and accessible medicine services.

**Patient or Public Contribution:**

The Patient and Public Involvement and Engagement (PPIE) group at Newcastle University had extensive input in the design and concept of this study before the research was undertaken. Throughout the work, a patient champion (Harpreet Guraya) had input in the project by ensuring that the study was conducted, and the findings were reported, with cultural sensitivity.

## 1 INTRODUCTION

Disparities within healthcare provision have been widely discussed in recent literature,^1^, ^2^, ^3^ with the coronavirus‐19 pandemic shedding further light on inequalities in access to healthcare across the globe.^4^, ^5^, ^6^ Healthcare inequalities and ethnicity are closely related, and yet, patients from ethnic minority groups have not been involved in health and social care research to the same extent as those from predominantly white groups.^7^ Evidence has shown that individuals from minority ethnic backgrounds report poorer general health when compared to their white counterparts.^8^ In addition, evidence has also demonstrated that patients from ethnic minority groups are more likely to report a long‐term illness than their white counterparts.^9^ Yet, these patients are reportedly less likely to engage in regular medicine reviews and have reported poorer adherence to prescribed medications to manage long‐term illness.^10^, ^11^

Regular reviews of patient medications, which include high‐quality information at the point of prescribing and considerations around deprescribing inappropriate medicines, are essential to support medicine effectiveness and prescribing safety.^12^, ^13^, ^14^ Medicine review services delivered in primary care settings (like New Medicine Services conducted by community pharmacists or structured medicine reviews performed by general practice pharmacists) are one method that exists within the United Kingdom's National Health Service (NHS).^15^, ^16^, ^17^ The focus of such services centres on improving the clinical effectiveness of medicines being taken, by addressing issues relating to medicine optimization and medicine adherence.^18^, ^19^, ^20^ The economic effectiveness of these interventions has also recently been explored, where Elliott et al.^21^ suggested that New Medicine Services would deliver better patient outcomes than normal practice at reduced costs to the health service in the long term. It is important to consider the *accessibility* of these services for patients to access and use these effectively, in particular, those patients from ethnic minority groups.^22^

Variation in healthcare access can be associated with social and cultural determinants creating inequality for ethnic minority patient groups.^23^ In previous medical literature across the globe, reduced access to healthcare for ethnic minority populations has been well reported,^8^, ^24^, ^25^, ^26^ and groups have been previously referred to as medically underserved across a range of health conditions.^27^, ^28^, ^29^ In recent studies, patients from minority ethnic groups were reported less likely to access and attend medicine‐based services.^30^ For example, Eh et al. found that Chinese immigrants who had stronger beliefs in the effectiveness of traditional Chinese medications were less likely to be adherent to prescribed medication^31^; a systematic review by Alhomoud et al. highlighted the paucity of research that examines medicine‐related needs for ethnic minority patient groups,^32^ and Latif et al. reported on the low level of evidence of underrepresented ethnic minority groups engaging in medicine‐related research,^33^ as well as the inequitable access to healthcare services, including pharmacies.^29^ Gaining knowledge of the barriers and facilitators that impact ethnic minority groups accessing medicine services is required to ensure that services are fit for purpose to meet and support the medicine‐centred needs of all patient populations.

In the United Kingdom, as demonstrated in other countries, the growth of various ethnic communities and linguistic groups, each with their own cultural traits and health profiles, presents a complex challenge to healthcare practitioners and policy makers in terms of achieving equitable access to healthcare. To shed light on the inequalities that impact the accessibility of medicine services, a greater understanding is required about the perceptions of healthcare professionals involved in the delivery of the services. By sharing the views of this cohort of healthcare professionals, this study aims to go beyond the existing patient‐centred research to better learn about ways to improve access for minority groups themselves. Limited studies are available that apply this lens, with even fewer focusing on the challenges of medicine‐specific contexts.^10^, ^25^, ^34^ This study investigates the details surrounding the barriers and facilitators to access for these patient groups and seeks to build on existing evidence to ask the following question: ‘What do healthcare professionals believe are the barriers and facilitators for patients of minority ethnic groups when accessing medicines services?’

## 2 MATERIALS AND METHODS

### 2.1 Recruitment and sampling

The consolidated criteria for reporting qualitative research (COREQ) checklist were followed for this study, according to EQUATOR guidelines (see Supporting Information File, Item 1).^35^ Immediately before study commencement, COVID‐19 restrictions were enforced across the United Kingdom. This meant that the planned face‐to‐face recruitment and data collection could no longer be undertaken in person. Instead, an amendment to University Ethics meant that participant recruitment could be conducted using remote methods. Purposive sampling was used to recruit participants from a range of healthcare professional groups, who were of mixed in terms of age ranges, ethnic backgrounds, clinical expertise and years of experience. Publicly available data were used to access email addresses, and all participants were invited to participate via an email invitation (which included a study information sheet and consent form). Participants who expressed an interest and provided their informed written consent were enrolled into the study. No prior relationship was established between the researcher and participants before study commencement or recruitment. Participants were also given the opportunity to ask questions before signing the consent form and were informed that they were free to withdraw from the study at any time. Inclusion criteria were as follows: participants working in a UK‐based healthcare professional role and those who perform medicine review services as part of their professional role.

### 2.2 Semistructured interviews

In depth, semistructured interviews were conducted by one researcher (M. E., a female undergraduate researcher with experience of qualitative research) between October and December 2020. Interviews were conducted with participants over the telephone or by using video call‐based software, such as Zoom® and Microsoft Teams®; all participants were offered the choice of which platform they prefer. The semistructured interview topic guide was developed based on two pilot interviews and covered key issues identified within the current literature focusing on ethnic minority groups (see the Supporting Information File, Item 2).^8^, ^24^, ^25^, ^36^ These issues included participants' experiences of performing medicine review services with ethnic minority patient groups and their perceptions of barriers and facilitators that may impact the service. Interviews were conducted until theoretical data saturation was reached, that is, upon author consensus that subsequent interviews yielded no new information.^37^, ^38^, ^39^

### 2.3 Data analysis

All interviews were audio‐recorded and transcribed verbatim by one researcher (M. E.). All data were anonymized at the point of transcription; participants did not provide comments on the transcripts or feedback on the results. A reflexive thematic analysis approach was performed by two researchers (M. E., a student pharmacist, and A. R., a pharmacist and qualitative researcher). Following reflexive thematic analysis processes, as defined by Braun and Clarke,^39^, ^40^ each interview was transcribed and analysed before conducting the next. Constant comparison guided an iterative process of data collection and analysis. Data familiarization was achieved through close and detailed reading of the transcripts. Initial descriptive codes were identified in a systematic manner across the data set. The codes were grouped into common coding patterns, which aided the development of analytic themes from the data. Themes were reviewed, refined and named once coherent and distinctive. If agreement was not reached between the two authors performing the data analysis, discussion and consensus were sought from the wider research team (A. H. and A. T., both experienced qualitative researchers and healthcare professionals). Interview field notes (maintained by M. E.) enhanced the reflective process. NVivo (version 12) software facilitated the data management processes throughout. When sharing participant quotes in this study, nonidentifiable pseudonyms have been used to ensure confidentiality, for example, Participant A, Participant B, and so forth.

### 2.4 Ethical approval

The study received full ethical approval from the Newcastle University Faculty of Medical Sciences Ethics Committee, with an ethical reference number of 5314/2020.

## 3 RESULTS

Eighteen participants were recruited and interviewed as part of this study (there were no refusals to partake, participant dropouts or repeat interviews). The characteristics of the healthcare professional participants are described in Table 1. The average age of the participants was 38 years (SD: 11.97), and the most common healthcare professional group interviewed was pharmacists (*n* = 9, 50%). Eleven interviews were conducted over the telephone and seven were performed using the video call‐based software, Zoom®.

**Table 1:** Participant characteristics

Participant	Sex (M/F)	Age range (years)	Interview format	Healthcare professional role	Ethnicity
1	F	60–69	Video‐call	Paediatric consultant	Indian
2	F	50–59	Telephone	GP pharmacist	White
3	F	30–39	Telephone	Registrar	Arab
4	F	50–59	Video‐call	Paediatrician	Arab
5	F	30–39	Telephone	GP pharmacist	Indian
6	F	30–39	Telephone	GP	Indian
7	F	30–39	Telephone	Registrar	White
8	F	20–29	Telephone	Hospital pharmacist	Black
9	F	30–39	Telephone	Community pharmacist	Pakistani
10	F	40–49	Video‐call	Hospital pharmacist	White
11	F	20–29	Video‐call	S/S pharmacist	White
12	M	40–49	Video‐call	GP	White
13	F	20–29	Telephone	Community pharmacist	Bengali
14	F	20–29	Telephone	Care home pharmacist	Arab
15	F	30–39	Video‐call	Hospital pharmacist	White
16	M	60–69	Video‐call	Respiratory consultant	Arab
17	F	20–29	Telephone	Dentist	White
18	M	30–39	Telephone	GP	White

Abbreviations: F, female; GP, general practitioner; GP pharmacist, pharmacist working in a GP surgery; M, male; Registrar, specialist doctor who has received advanced training in a specialist field of medicine; S/S pharmacist, split sector pharmacist (working in hospital and a GP surgery).

**Figure 1:**
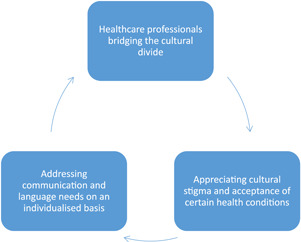
Barriers and facilitators that affect access to medicine services for ethnic minority patients

Three key themes were developed from the data to highlight the perceived barriers and facilitators that affect access to medicine review services for ethnic minority patients. These themes centred around (1) healthcare professionals bridging the cultural divide; (2) appreciating cultural stigma and acceptance of certain health conditions; and (3) addressing communication and language needs on an individualized basis (as demonstrated in Figure 1). Each of these themes is discussed in turn and participant perspectives and recommendations are shared throughout as anonymized interview quotes.

### 3.1 Theme 1: Healthcare professionals bridging the cultural divide

Participants discussed how certain cultural expectations and fundamental understanding of what the UK healthcare system provides can pose as barriers to accessing care. One general practitioner (GP) described their understanding as follows: ‘general practice isn't a specialty that exists in every country, so some people don't really know our role’ (Participant 18). They reflected that patient expectations of health services can be better understood when healthcare professionals have appreciation of other cultures. Cultural competency awareness can act as a facilitator to remove this barrier and support patient understanding to get the most out of their healthcare. One participant stated ‘making sure if somebody prefers a female GP, there is a female GP available and just being more culturally aware’ (Participant 12). They also felt that, although healthcare professionals are ‘generally more aware of (differences in cultures) than they were in the past’, they recognized ‘that it is still a barrier for some patients’ (Participant 12).

if they (healthcare professionals) are struggling to understand that person, or if they don't understand their background, then they are not going to cater their treatment towards what's best for them… it would be good to have teaching sessions about different cultures… ethical dilemmas and how different faiths and different ethnicities might deal with these. (Participant 14)

By improving patient familiarity with the NHS and health services, barriers to accessibility and unmanaged expectations can be overcome. One participant discussed the importance of recognizing those patients who ‘are unlikely to be familiar with the NHS’ and providing additional support if ‘they don't know how to access services, and maybe don't know how prescription system works or how to go to the pharmacy’ (Participant 12). Factors such as ‘how long they've been in the UK, whether they were born in the UK and whether they recently arrived in the UK’ were all discussed as barriers that may impact a person's understanding and expectations relative to their health (Participant 12).

One participant also discussed a wider, system‐level approach to overcome accessibility barriers for patients of ethnic minorities. Perceptions were shared about the need to separately view ‘patients who have arrived here within the last two or three years or asylum seekers’ from those who ‘have grown up here or their families have grown up here’, on the basis that the ‘key difference is whether or not you understand how the system works and how to access medicines services’ (Participant 12). By appreciating the immigration history of individual patients, healthcare professionals can work to break the barriers relating to the accessibility of medicine review services and strive for equality. By gaining an understanding of a person's cultural beliefs and expectations, healthcare professionals and policy makers can ensure the design and delivery of medicine review services that are culturally acceptable and adaptable.

if you've only recently arrived then you might be used to a different healthcare system, maybe either a private healthcare system or no healthcare system, depending where you come from. (Participant 12)

### 3.2 Theme 2: Appreciating cultural stigma and acceptance of certain health conditions

Participants shared their perceptions of barriers to accessing medicine services that exist as a consequence of cultural beliefs of ethnic minority communities, in particular, the diagnosis and treatment of certain physical and mental health conditions. One participant reflected on their prior experience of ‘giving bad news… it's lung cancer’ to minority ethnic patients from the Middle East. This participant felt that ‘people from the Middle East, it's very difficult for them to accept such bad news… to a degree (where) sometimes the families are trying to protect the patient and “say don't tell him the diagnosis”’, which, in turn, affected the patient's engagement with treatment (Participant 16). Another participant, who specialized in paediatric health, discussed similar experiences relating to barriers that exist from the cultural beliefs of accepting a diagnosis and treatment for a child with Autism. This participant described seeing this ‘more in ethnic minorities… they are not acknowledging that problem of their child’ (Participant 4). In particular, the participant discussed the impact that cultural barriers can have around the ‘stigma that accompanies’ a diagnosis, as well as the traditional cultural beliefs that ethnic minority groups may have towards certain health conditions. As a consequence, access to appropriate medicines and care were delayed.

a parent who refused my diagnosis that the child has Autism… the mum requested not to share it with the school and to take the diagnosis off (the child's medical records). I said I can't take the diagnosis off because it has been done, what I can do is not to share the letter with the school it's your right… and if you change your mind, I'm quite happy to see you again. What happened is six months later, because the school refused to give the right support, because he doesn't have the right diagnosis, the mum came and asked for help. So, the challenges are sometimes the cultural beliefs. (Participant 4)

In a similar way, participants reflected on a cultural barrier that has impacted discussions on mental health conditions within ethnic minority communities, when compared to their white counterparts. One participant, who worked as a GP in a highly populated Romanian and South‐East Asian area, described seeing patients from ethnic minorities ‘suppress their symptoms’ of depression and anxiety due to cultural beliefs of shame and embarrassment (Participant 6). This participant went on to explain how these cultural behaviours can later manifest as generic physical pain, which is more difficult to treat and manage.

(patients) often go to see a GP and say ‘I've got aches and pains here, I've got this, I've got that’ like there's always non‐specific symptoms… depression, anxiety—they aren't really recognised, they just say that you need to crack on, it doesn't really exist. What they often do is suppress the symptoms… they're not recognizing it. (Participant 6)

Similar views were shared by participants working in other healthcare specialties, where it was recognized that age and underlying cultural beliefs may play a role in acceptance and support‐seeking when it came to a person's mental health. One participant, with experience of working as a GP for asylum seeker groups, stated that ‘older patients are less likely to come to the GP… I'm thinking particularly around mental health (where) older patients would be less likely to see the GP compared to younger patients… I'm thinking specifically in an older, immigrant population’ (Participant 12). From their experience, this participant stated that patients from ethnic minority groups were ‘less likely to present with a mental health problem but actually, sometimes they come with a physical problem and when you dig down into it, you realise that there's mental health problems underlying it… and there's an under‐diagnosis of dementia and cognitive problems in some minority populations too’ (Participant 12). Another reason given for this was associated with thoughts of ‘other causes for their mental health’ symptoms, often rooted in religious or spiritual beliefs that would not respond to medical treatment (Participant 12).

I'm thinking about a patient from Malawi, where they had um… particular sort of symptoms that they and their family had attributed to witchcraft or just spiritual beliefs… (if) my symptoms are caused by witchcraft then a tablet isn't going to fix that problem… or like, seeing a therapist isn't going to fix that problem. (Participant 12)

### 3.3 Theme 3: Addressing communication and language needs on an individualized basis

All healthcare professionals who were interviewed remarked on the existing barriers of communication and language when patients from ethnic minority groups access medicine services. Participants discussed the need for inclusive medicine‐specific resources, like leaflets, that could be produced in multiple languages to overcome barriers and better support patient understanding about their medicines. One participant reflected on their experience with language and communication barriers whilst working as a GP for asylum seekers, and reported ‘not being able to access other resources that are available (is) obviously a problem from the NHS… we don't provide resources in a wide range of languages, and we expect that patients will be able to educate themselves about things’ (Participant 12). Another GP discussed steps already being taken in their locality around communicating ‘public health messages amongst the communities, which we do try and do here in [name of city] for different community groups… making sure it's published in lots of different languages’ to bridge communication gaps (Participant 18).

Limitations in communication also meant that some participants felt that they were unable to give the full medication counselling or advice, compared to the amount they would provide to patients who were fluent in English. Participants considered this to negatively impact medicine issues including prescribing safety and poor treatment adherence, if patients were not fully aware of the medicine rationale and importance of medicine‐taking.

If I said to someone you need to take that daily, ‘just one daily’, then that's it. That's different to if I had a conversation with them explaining ‘why you need to take that, if you don't take that then this is the route you are looking at, if you do take it you are looking at a better quality of life’… making the patient understand from your perspective, getting them to see a long‐term picture. If you've got a communication barrier, you can't take them through that (Participant 9)

Another participant acknowledged the importance of wider messages of communication across cultures, where the way in which symptoms are described in one culture may not translate into another community or language.

Sometimes the way that we describe symptoms… we have a particular way, and maybe in the UK or in Europe describing certain some mental health symptoms… (the descriptions) they maybe don't translate into the communities or other languages. You know when we talk about mental health, we use lots of euphemisms and lots of idioms, and they can be mistranslated and maybe people from a different background have a different understanding of how to describe their symptoms… it can get lost (Participant 12)

Other means of translation, including NHS‐based interpreter services, were widely discussed in the interviews. The availability of interpreter services appeared to vary between healthcare settings and locations across the United Kingdom. While some participants praised current services where, ‘if you need an interpreter, they can generally be readily available’ (Participant 14), other participants discussed difficulties in service availability or not meeting the individual needs of patients. One hospital registrar stated that ‘if the language is a very specific dialect… it's very hard to get someone to translate’ (Participant 7). Another participant reflected on the impact that interpreter services can have when facilitating a person‐centred consultation, where ‘all of your conversation should be directed to the patient… but when it goes to a third party that makes the interaction probably not as personable as it would be otherwise’ (Participant 10).

Some participants reflected on strategies that they have used in attempts to bridge the language gap, if interpreters were unavailable. Reliance on ‘a family member who understands English. (I) communicate with them and they can translate for me’ was often discussed (Participant 8). Participants acknowledged the integral role that family members can play within minority ethnic cultures. In their experience of working as a pharmacist conducting medicine reviews, one participant discussed benefits for patients of ‘South Asian background or Arab background… (they are) more likely to have more input from their children and so, sometimes, actually it makes it a lot easier to do things like medicines reconciliations because you know there's someone there who is looking after them and so you can go through (the medicines) with them’ (Participant 14). However, in certain instances, participants believed translation by family members to be inappropriate, for instance, ‘if it's a medicine for an intimate or sensitive subject, they may not be too keen to discuss that with a family member’ (Participant 2).

Clear communication about medicines was recognized as a significant barrier by participants; however, facilitators to overcome this were also considered. One participant described the use of digital translator devices in their community pharmacy‐based consultations, where ‘Google translate’ was previously used ‘so that I know, whatever I'm saying, they're understanding it properly’ if they or other staff members did not speak the same language as the patient (Participant 13). A hospital pharmacist stated that they ‘found this pharmacy website that created a label in the language that the patient speaks’ as a specific way to support ethnic minority patients being discharged from hospital (Participant 8). This participant also discussed the development of ‘NHS approved’ digital translator technologies that could offer practitioners the same degree of flexibility and ease when discussing and reviewing a patient's medicines, while being more reputable than other digital alternatives (Participant 8).

Accounting for additional time pressures in appointments for patients with translation requirements was a recognized facilitator to manage communication and language barriers. Participants raised concerns that adequate time was often not allocated to adequately address patient needs. One GP spoke about strategies implemented within their surgery to facilitate appointments for ethnic minority patients with translator requirements, thus supporting meaningful medicine‐specific discussions.

We try to book a double appointment if we had an interpreter… if they've got limited English, it's difficult for them to access services, so when they do, then they often have lots of problems that they want to discuss… I would want to know that I've got enough time to deal with the issues properly. (Participant 12).

## 4 DISCUSSION

This study builds on the limited evidence base that focuses on healthcare professional perspectives when delivering medicine reviews for patients from ethnic minority groups. By collecting the perspectives of healthcare professionals who are involved in the delivery of medicine reviews, this study sheds light on the existing barriers and enablers that affect service accessibility in a bid to improve the quality and delivery of culturally competent, person‐centred care across all patient populations.

A consistent finding across interviews was that participants identified differences in cultural backgrounds between healthcare professionals and patients to be a key contributor to inequalities when accessing healthcare and medicine‐specific services. A lack of cultural understanding and cultural competence by healthcare professionals can have severe implications for patient care. This is echoed by previous work^24^, ^41^ that instils the importance of recognizing that individuals have diverse identities. To achieve culturally competent care, the cultures of care recipients and healthcare providers are of significance.^25^ Involving the patient and the healthcare professional in coproduction approaches may be a useful strategy to overcome this barrier and develop and refine a targeted intervention to support access to medicine services for minority ethnic patients. It is apparent that, to be of the best use for patients and professionals, any such intervention should be designed with a holistic, culturally sensitive and culturally competent approach.^10^

Results from this study echo previous studies in demonstrating that certain health conditions, in particular mental health issues, still carry a stigma within ethnic minority communities that causes barriers to seeking medical help and adhering to medication‐based treatment.^42^, ^43^ The EMPIRIC study by Weich et al. demonstrated that particular ethnic groups (middle‐aged Irish and Pakistani men, and older Indian and Pakistani women) had significantly higher rates of mental health disorders than their white counterparts.^44^ The 2007 adult psychiatric morbidity survey also found that individuals from nonwhite ethnicities were less likely to consult healthcare professionals about their mental health and thus less likely to be prescribed medications like antidepressants to manage their symptoms.^45^ Ethnic minority patients were also more likely to be diagnosed with severe mental illness and, therefore, receive higher doses of medication.^46^ Factors such as cultural, religious or spiritual beliefs should be considered and better integrated into clinical assessments.^47^ For instance, developing a greater awareness of potential cultural barriers that may arise with patients from ethnic minority groups should be a priority.^48^, ^49^, ^50^, ^51^, ^52^, ^53^ Thus, healthcare professionals may be better equipped with strategies to overcome them to facilitate better and equal access to appropriate medicine review and supportive care.^48^, ^54^, ^55^, ^56^, ^57^

Communication is an important prerequisite for successful healthcare access and outcomes.^58^ Due to the presence of a language barrier, studies have reported that ethnic minority patients with limited English demonstrate lower rates of medication adherence, make fewer visits to healthcare professionals, have reduced understanding of their conditions and treatment and develop increased medical complications.^11^, ^59^ Language barriers mean that patients may lack the ability to express themselves and may feel embarrassed to seek medical advice, further hindering the ability to communicate and build a rapport between the healthcare professional and the patient.^60^, ^61^ The use of an independent translator could circumvent the language barrier issue.^62^ The study by Karliner et al. found that professional interpreters were associated with improved quality of care and reduced differentials in access to care.^63^ Participants in this study also cited the importance of translators as a facilitator for minority patient access; however, they also cited the challenges associated with accessing interpreters in a timely manner to support consultations. The lack of availability of interpreters in pharmacy‐based settings has been a recognized source of patient safety concern in the wider literature,^64^, ^65^ with Chauhan et al. reporting low English‐language proficiency as a contributor to increased risk of patient safety events for ethnic minority populations.^59^ In the absence of interpreters, participants of this study discussed reliance on other methods to communicate, such as Google® translate. In other studies, such situations were associated with limited quality‐of‐care communications and a lack of information transfer, including medication changes and counselling.^66^, ^67^ By encountering a language barrier where an interpreter may also not be available, healthcare professionals could find themselves providing the patient with limited information regarding their medication in the scope that the patient can understand. As a result, this may lead to reduced condition comprehension, medicine adherence and a poorer long‐term prognosis.^68^, ^69^ The opportunity to use written multilingual prescription labels has been researched in the recent literature^70^; however, considerations should be made for those patients who communicate in non‐English languages verbally, rather than in written format.

Eighteen participants were purposively sampled and interviewed in this study to include a range of experiences across different roles and settings, who routinely engage in the provision of medicine services. However, we acknowledge that there are some limitations in this study. The addition of other healthcare professional groups (such as nurses) may have provided additional insight. Many participants themselves were from minority ethnic backgrounds, and thus were able to provide in‐depth, first‐hand insight with cultural appreciation. There was no representation from East Asian participants, which may have conveyed alternative perspectives in the data. The intended method of in‐person data collection was impacted by the COVID‐19 pandemic. Although video call‐based software can replicate face‐to‐face interviews in the ability to respond to verbal and nonverbal cues,^71^, ^72^ there are some disadvantages to note with this interview technique.^73^ User familiarity and comfort of use may have resulted in the higher number of telephone‐based interviews with participants.^74^ This study includes the perspectives of healthcare professionals involved in the delivery of medicine services to patients from ethnic minority groups. What remains to be better examined is the perceptions of patients themselves, to understand their lived experiences of accessing medicine services. Future studies should seek to utilize coproduction approaches that involve patients from underrepresented ethnic minority groups alongside healthcare professionals.^75^ Latif et al. described coproduction approaches as a reflective opportunity for community pharmacy professionals to review services offered to medically underserved groups, including those from ethnic minority backgrounds.^33^ Previous studies have also implemented coproduction approaches to tailor health services to the needs and preferences of service users.^76^, ^77^, ^78^ Done in partnership with patient representatives from the communities being researched, coproduction can better extend the understanding of the lived experiences of ethnic minority groups in terms of accessibility. As a result, further investigation may enable the recognition and resolution of barriers and facilitators that would enable improved accessibility and inclusivity for ethnic minority communities.

## 5 CONCLUSION

Acknowledging the barriers and facilitators to ethnic minority groups is an important step towards ensuring equality in access to medicine services. Before this study, limited data existed that explored the perspectives of healthcare professionals involved in delivering medicine review services, particularly in relation to barriers and facilitators affecting ethnic minority patient access. This study seeks to address this gap and provides much‐needed evidence implicating the delivery of person‐centred care and considering changes based on a systems‐level and an individualized person level. Coproduction approaches should be adopted to support better understanding of ethnic minority cultures and thus inform the design and delivery of culturally sensitive, medicine review services. Findings from this qualitative study should be used alongside patient‐informed research to work to achieve equal access to medicine services for all.

## CONFLICT OF INTERESTS

The authors declare that there are no conflict of interests.

## Supporting information

Supporting information.

## Data Availability

The data that support the findings of this study are available from the corresponding author upon reasonable request.
