# Supplementary material for: A qualitative exploration of the barriers and facilitators affecting ethnic minority patient groups when accessing medicine review services: Perspectives of healthcare professionals
Source: Health Expect. 2021 Dec 23;25(2):628–38. doi: 10.1111/hex.13410 (PMC8957739; doi:10.1111/hex.13410)
Supplement: Supplementary file 1 — Supporting information. [file HEX-25--s001.docx]

**Item 1:**

**COnsolidated criteria for REporting Qualitative studies (COREQ): 32-item checklist.**

| **Number** | **Item** | **Guide questions / description** | **Reported on manuscript page** |
| --- | --- | --- | --- |
| **Domain 1: research team and reflexivity** | | | |
| **Personal characteristics** | | | |
| 1 | Interviewer | Which author(s) conducted the interviews? | 7 |
| 2 | Credentials | What were the researcher’s credentials? *E.g., PhD, MD* | 7 |
| 3 | Occupation | What was their occupation at the time of the study? | 7 |
| 4 | Gender | Was the researcher male or female? | 7 |
| 5 | Experience and training | What experience or training did the researcher have? | 7 |
| **Relationship with participants** | | | |
| 6 | Relationship established | Was a relationship established prior to study commencement? | 6 |
| 7 | Participant knowledge of interviewer | What did the participants know about the researcher?  *E.g., reason for doing the research* | 6 |
| 8 | Interviewer characteristics | What characteristics were reported about the interviewer?  *E.g., bias, assumptions, reasons and interests in the research topic* | 7 |
| **Domain 2: study design** | | | |
| **Theoretical framework** | | | |
| 9 | Methodological orientation and theory | What methodological orientation was stated to underpin the study?  *E.g., grounded theory, ethnography, discourse analysis* | 7 |
| **Participant selection** | | | |
| 10 | Sampling | How were participants selected? *E.g., purposive, convenience, consecutive* | 6 |
| 11 | Method of approach | How were participants approached? *E.g., face-to-face, telephone, email* | 6 |
| 12 | Sample size | How many participants were in the study? | 8 |
| 13 | Non-participation | How many people refused to participate or dropped out (with reasons)? | 8 |
| **Setting** | | | |
| 14 | Setting of data collection | How was the data collected? *E.g., home, clinic, workplace* | 7 |
| 15 | Presence of non-participants | Was anyone else present besides the participant and researcher? | 7 |
| 16 | Description of sample | What are the important characteristics of the sample? *E.g., demographic data* | 8 |
| **Data collection** | | | |
| 17 | Interview guide | Were questions and prompts provided by the authors? | 6-7 |
| 18 | Repeat interviews | Were repeat interviews carried out? If yes, how many? | 8 |
| 19 | Audio/visual recording | Did the researcher use audio or visual recording to collect the data? | 7 |
| 20 | Field notes | Were field notes made during/after the interview? | 8 |
| 21 | Duration | What was the duration of the interviews? | 8 |
| 22 | Data saturation | Was data saturation discussed? | 7 |
| 23 | Transcripts returned | Were transcripts returned to participants for comment/correction? | 8 |
| **Domain 3: analysis and findings** | | | |
| **Data analysis** | | | |
| 24 | Number of data coders | How many data coders coded the data? | 7 |
| 25 | Description of the coding tree | Did authors provide a description of the coding tree? | N/A |
| 26 | Derivation of themes | Were themes identified in advance or derived from the data? | 7 + 9 |
| 27 | Software | What software, if applicable, was used to manage the data? | 7 |
| 28 | Participant checking | Did participants provide feedback on the findings? | 7 |
| **Reporting** | | | |
| 29 | Quotations presented | Were participant quotations presented to illustrate the themes / findings? Was each quotation identified? E*.g., participant number* | 9-16 |
| 30 | Data and findings consistent | Was there consistency between the data presented and the findings? | Yes |
| 31 | Clarity of major themes | Were major themes clearly presented in the findings? | Yes |
| 32 | Clarity of minor themes | Is there a description of diverse cases or discussion of minor themes? | Yes |

**Item 2:**

**Semi-structured interview topic guide**

The semi-structured interview questions were based around the following topic areas:

1. Description of job role and interaction with patients
2. Experience of delivering medicine review services
3. Experience of treating patients from ethnic minority groups
4. Perceived similarities, differences, barriers and facilitators to medicine review services for ethnic minority patients
